# Timing of administration of prophylactic antibiotics for caesarean section: a systematic review and meta-analysis

**DOI:** 10.1111/1471-0528.12036

**Published:** 2012-11-06

**Authors:** H Baaqeel, R Baaqeel

**Affiliations:** a College of Medicine–Jeddah, King Saud bin Abdulaziz University for Health Sciences and Department of OB/GYN, King Abdulaziz Medical City-WRJeddah, Saudi Arabia; b St Joseph's Health Centre, University of Western OntarioLondon, ON, Canada

**Keywords:** Caesarean section, intraoperative, preoperative, prophylactic antibiotics, timing

## Abstract

**Background:**

Prophylactic antibiotics reduce infectious morbidity from caesarean section. The timing of their administration, however, is a matter of controversy.

**Objectives:**

To examine maternal and neonatal infectious morbidity in women receiving preoperative prophylaxis compared with those receiving intraoperative administration.

**Search strategy:**

Medline, Embase, Current Controlled Trials and Cochrane Central were searched from their inception dates to December 2011.

**Selection criteria:**

Randomised controlled trials of a single dose of any antibiotic comparing preoperative with intraoperative administration were selected.

**Data collection and analysis:**

Trial characteristics, outcomes and quality measures, based on the Cochrane tool for risk of bias, were independently extracted. The random effect model of DerSimonian and Laird to estimate relative risks (RRs) for maternal and neonatal outcomes was used.

**Main results:**

Six trials met the inclusion criteria, reporting on 2313 women and 2345 newborns. Preoperative administration was associated with a significant 41% reduction in the rate of endometritis compared with intraoperative administration (RR 0.59; 95% confidence interval [95% CI] 0.37–0.94; *I*^2^ 0%). In the preoperative group, there were nonsignificant reductions in the rates of wound infection (RR 0.71; 95% CI 0.44–1.14; *I*^2^ 0%), maternal febrile morbidity (RR 0.94; 95% CI 0.46–1.95; *I*^2^ 0%), neonatal sepsis (RR 0.81; 95% CI 0.47–1.41; I^2^ 0%), neonatal septic work-up (RR 0.93; 95% CI 0.71–1.21; *I*^2^ 0%) and neonatal intensive-care unit admission (RR 0.92; 95% CI 0.65–1.28; *I*^2^ 0%). There were nonsignificant increases in the rates of maternal pyelonephritis (RR 1.09; 95% CI 0.49–2.43; *I*^2^ 0%) and neonatal pneumonia (RR 3.36; 95% CI 0.55–20.47; *I*^2^ 0%).

**Conclusions:**

Compared with intraoperative administration, preoperative antibiotics significantly reduce the rate of endometritis. The lack of neonatal adverse effects should be cautiously interpreted given the limited power of the trials to detect such effects.

## Introduction

### Rationale

The beneficial effect of prophylactic antibiotics in reducing the occurrence of infectious morbidity from caesarean section, whether elective or emergency, is well established.^1^ A single dose of first-generation cephalosporin is as effective as multiple doses of broad-spectrum agents.[Bibr b2] Prophylactic antibiotics for caesarean section are commonly used worldwide, and in most institutions a single dose is administered, generally after clamping of the umbilical cord.^3^,^4^ However, a recent survey (published in 2011) of maternal and fetal medicine physicians in the USA revealed that 84% of those who responded (the response rate was 25%) used preoperative administration.^5^ The effectiveness of prophylactic antibiotics depends on their presence in effective concentrations throughout the operative period. Classen et al.^6^ found that administration of prophylactic antibiotics within a 2-hour period preoperatively was associated with the lowest surgical wound infection rate. Because of concerns about unnecessary fetal exposure, masking of fetal infection, increases in neonatal septic work-up and the emergence of resistant strains when prophylactic antibiotics are given preoperatively, it is a common obstetric practice to administer prophylactic antibiotics after cord clamping. After administration of a 1-g preoperative dose (0.5–6 hours) for elective caesarean section, the cefazolin concentration in maternal and fetal blood, as well as amniotic fluid, was found to be equal to or greater than the mean concentration inhibiting 90% of group B streptococcus strains.^7^ Concerns about the increasing use of intrapartum antibiotic prophylaxis (IAP) to prevent neonatal group B streptococcal sepsis, particularly in North America, has led to some publications on the neonatal impact of such practices. The use of IAP was associated with reduced initial bacterial colonisation by *Clostridium* in the antibiotic-exposed infants, and did not promote colonisation by β-lactam-resistant bacteria.^8^ Infants whose mothers received IAP were not more likely to undergo invasive procedures or to receive antibiotics.^9^ Conflicting results were reported for neonatal sepsis. Two studies, a large historical cohort study of 17 187 infants and a small case–control study of 132 infants, reported similar rates of neonatal sepsis among IAP-exposed and non-exposed neonates,^10^,^11^ whereas two other studies, a retrospective review of 35 women and a study in which 27 women were prospectively followed, showed an increased rate of non-streptococcal organisms resistant to ampicillin in neonates exposed to IAP compared with those who were not exposed.^12^,^13^ *In utero* exposure to antibiotics has been linked to development of allergic diseases in infancy.^14^ Long-term effects of fetal exposure to antibiotics are beyond the scope of this review. Given that preoperatively administered antibiotics are associated with the lowest rate of surgical site infection and that, from a practical point of view, administration of the antibiotic preoperatively is less likely to be overlooked than after cord clamping, especially if intraoperative complications develop, preoperative administration seems more rational. The conflicting reports about the neonatal impact of the timing of prophylactic antibiotic administration prompted us to undertake this systematic review. While our review was in preparation, Costantine et al.^15^ published a systematic review on the timing of the administration of prophylactic antibiotics in caesarean section. Our review updates the information contained in the Costantine et al. review.

### Objectives

To examine whether there are differences in the rates of maternal and neonatal infectious morbidity, we reviewed randomised controlled trials that compared a single dose of any antibiotic administered preoperatively with a single dose of the antibiotic administered intraoperatively at or after clamping of the umbilical cord in women undergoing caesarean section.

## Methods

### Protocol and registration

The search strategy, inclusion criteria and methods of analysis were specified in advance in a protocol that used the Cochrane Collaboration format but was not registered with any entity. The PRISMA Statement^16^ was followed in this review.

### Eligibility criteria

The eligibility criteria for the inclusion of work in the review were as follows.

Type of study: randomised controlled clinical trials investigating the timing of administration of prophylactic antibiotics in women undergoing caesarean section.

Type of participants: women undergoing any type of caesarean section, whether elective or during labour, were considered.

Type of intervention: trials comparing a single dose of any antibiotic administered preoperatively with a single dose of the same antibiotic administered intraoperatively at or after clamping of the umbilical cord.

Type of outcome measures: maternal outcomes included febrile morbidity, endometritis, wound infection and pyelonephritis. Neonatal outcomes included neonatal sepsis, neonatal septic work-up and neonatal intensive-care unit (NICU) admission. Trials that reported one or more of these outcomes were considered.

### Information sources

Electronic databases that we searched (June 2007) included Medline (1966 to present), the Pre-Medline database, Embase (1980 to present), Current Controlled Trials and Cochrane Central. We also screened the reference lists of included trials and related review articles. No language or publication date limits were imposed.

### Search

We used the following search terms and corresponding index terms: caesarean, cesarean, caesarean section, cesarean section, caesarean delivery, cesarean delivery, abdominal delivery, antibiotics, antimicrobials, prophylaxis and prophylactic antibiotics. The operators ‘OR’ and ‘AND’ were used to combine these terms. The final results were limited to clinical trials. An example of a midline search strategy is shown in the Supplementary material, Appendix S1.

### Study selection

Both authors screened the titles and abstracts of the identified studies independently to exclude duplicates, letters to editors, editorials, review articles, retrospective studies and studies unrelated to the review question. The full text of the studies was obtained when titles or abstracts did not contain enough information. Remaining potentially eligible studies were retrieved for detailed evaluation. Eligibility criteria were applied independently by both authors to identify final eligible studies. Disagreement was resolved by discussion.

### Data collection process

Each reviewer, independently, extracted data from each included study using a predesigned data extraction form. Discrepancies were resolved by both authors checking the study against the form. Authors were contacted for missing data.

### Data items

Information was extracted from each study on: (1) characteristics of the participants, including type of caesarean section and the inclusion and exclusion criteria of the trial; (2) the type of intervention, including the type, dose, route of administration and timing of antibiotic administration; (3) the type of outcome measures, with their definition as reported in each study, including maternal febrile morbidity, wound infection, endometritis, pyelonephritis, neonatal sepsis, neonatal septic work-up, NICU admission and other outcomes reported in the trials.

### Risk of bias in individual studies

To assess the risk of bias in the included studies, both authors independently determined the adequacy of randomisation, concealment of allocation and blinding, and the extent of loss to follow up. Any disagreement was resolved by discussion.

### Summary measures

Quantitative analyses were performed by computing relative risks (RRs) and their 95% confidence intervals (95% CIs) for dichotomous outcome variables. For continuous outcome variables a mean difference was calculated.

### Synthesis of results

A pooled estimate of outcomes was computed using the more conservative DerSimonian and Laird random effects model. We tested for statistical heterogeneity using the conventional Cochrane's *Q* test which is based on the chi-square test, *I*^2^ and tau-square tests. The quality of evidence for each outcome was graded using GradePro.^17^

### Risk of bias across studies

The possibility of publication bias was assessed, when applicable, using a funnel plot of the effect of each trial against its standard error, and any asymmetry was checked using an adjusted rank correlation test^18^ and the regres sion-based assessment of this asymmetry as described by Egger et al.^19^ All analyses were performed using Review Manager 5,^20^ and for publication bias tests Meta-analysis Made Easy (MIX) was used.^21^,^22^

### Additional analysis

Sensitivity analysis was performed if a study was determined to have an intermediate or high likelihood of bias that may influence the pooled estimates of the outcome of interest.

## Results

### Study selection

**Figure 1:**
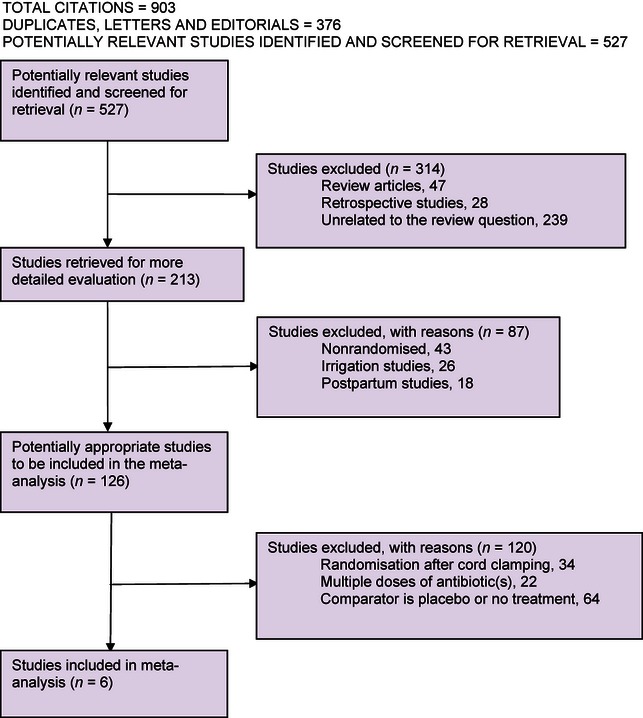
Study selection flow chart.

The combined yield of the search of all the sources was 903 citations. Initial screening identified a total of 376 as duplicates, letters or editorials. Figure 1 depicts a flow chart for study selection from the remaining 527 citations. An ongoing trial was identified through the National Institute of Health's Clinical Trials Registry that was registered in August 2005 but terminated in September 2006 without publishing any report.[Bibr b23] Six studies met the inclusion criteria so were included in the meta-analysis.^24^–^29^

### Study characteristics

All six studies were randomised controlled trials. Four studies were from the USA, one was from Turkey^27^ and one was from Austria.^29^ Two studies were limited to emergency caesarean section^24^,^25^ and the remaining four were limited to elective caesarean section^26^–^29^. Four of the studies used 1 g cefazolin and two used 2 g.^25^,^29^ In all the studies the primary outcome of maternal infectious morbidity was used for the power calculation. Table 1 shows the characteristics of the included studies.

**Table 1:** Characteristics of included studies

Source	No. of women	Type of CS	Primary outcome	Exclusion criteria	Antibiotic agent
Wax et al. 24	90	Emergency	Endometritis or wound infection	Allergy, antibiotic use within 2 weeks, temperature ≥37.8°C, prophylaxis for GBS or SBE infections, IDDM, HIV infection, chronic glucocorticoid use, multiple gestation and gestation <37 weeks	Cefazolin 1 g
Thigpen et al. 25	302	Emergency	Endometritis or wound infection	Allergy, antibiotic use within 2 weeks and acute chorioamnionitis	Cefazolin 2 g
Sullivan et al. 26	357	Elective	Total maternal infectious morbidity	Allergy, antibiotic use within 1 week, age <18 years and gestational age <24 weeks	Cefazolin 1 g
Yildirim et al. 27	389	Elective	Postoperative infections	Antibiotic use within 24 hours, fever on admission, DM, collagen vascular disease, immune system problems, chorioamnionitis, need for blood transfusion, ROM and gestational age <37 weeks	Cefazolin 1 g
Macones et al. 2011	434	Elective	Composites of maternal infection	Known fetal anomalies, exposure to antibiotics within 7 days, emergency CS, ROM greater than 18 hours and overt intrapartum infection requiring antibiotics	Cefazolin 1 g*
Witt et al. 29	741	Elective; ROM and uterine contractions were allowed	Postoperative infections	Fever greater than 38°C, cephalosporin allergy, age <18 years and exposure to any antibiotic agent within 1 week before delivery	Cefazolin 2 g**

CS, caesarean section; DM, diabetes mellitus; GBS, group B streptococcus; HIV, human immunodeficiency virus; IDDM, insulin-dependent diabetes mellitus; ROM, rupture of membranes; SBE, subacute bacterial endocarditis.

* Subjects allergic to penicillin received clindamycin 900 mg. Over 90% were reported to have received cefazolin.

** The trial had a third placebo arm.

### Risk of bias within studies

Assessment of risk of bias within studies is shown in Table 2. The included trials were of good methodological quality except for the trial by Yildirim et al.^27^ Insufficient details were given to enable assessment of blinding at all levels. The authors stated:

Four hundred women who fulfilled the inclusion criteria were randomly (two parts, block random using sealed, sequentially distributed envelopes to which the letters A and B had been allocated: the letter A to the antibiotic prophylaxis before skin incision group and the letter B to the antibiotic prophylaxis after clamping umbilical cord group; the patients chose the envelopes which were opened by the investigator, and according to the letters, the group of patients were determined) divided into two groups.

**Table 2:** Risk of bias within studies

Trial	Randomisation		Blinding				Loss to follow-up
			
	Method	Concealment	Patient	Provider	Data collector	Outcome assessor	
Wax et al. 24	Computer-generated	Yes	Yes	Yes	Yes	Yes	6/90 (6%) at 2 weeks 14/90 (15%) at 6 weeks
Thigpen et al. 25	Computer-generated	Yes	Yes	Yes	Yes	Unclear	44/346 (13%) at 6 weeks
Sullivan et al. 26	Random tables	Yes	Yes	Yes	Yes	Unclear	8/357 (2%) at 6 weeks
Yildirim et al. 27	No details except ‘two parts block randomisation’	Yes	Insufficient details. Probably no.				11/400 (3%) at 6 weeks
Macones et al. 2011	No details except ‘permuted blocks’	Probably yes	Yes	Yes	Yes	Yes	None
Witt et al. 29	Computer-generated	Yes	Yes	Yes	Yes	Yes	32/741 (4.3%) at 4 weeks

From the published report, it appears that there was no blinding.

### Results of individual studies

**Figure 2:**
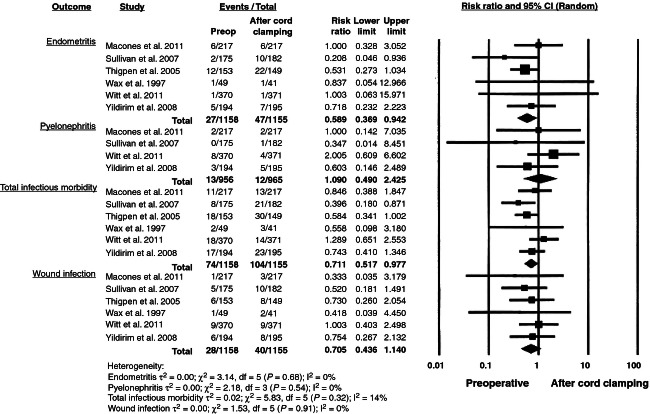
Forest plot showing results of the meta-analysis for maternal outcomes.

**Figure 3:**
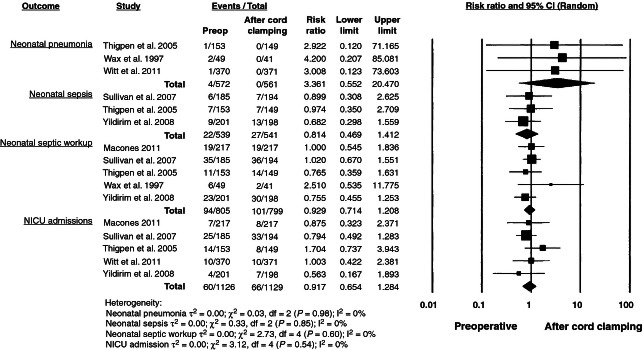
Forest plot showing results of the meta-analysis for neonatal outcomes.

The six included trials recruited a total of 2313 women and 2345 neonates. The definitions of outcomes were basically similar in all trials when reported. The breakdown of neonatal outcome was not sufficiently detailed in two trials.^28^,^29^ One trial reported outcomes in percentages or mean values and lumped suspected cases of sepsis together without reporting how many had been confirmed. The other trial reported that neonatal outcomes were similar among groups without providing actual rates. The corresponding authors were contacted. The missing data were provided by one of them.^29^ Individual trial results in a forest-plot format are shown for the outcomes endometritis, wound infection, pyelonephritis and total maternal infectious morbidity in Figure 2, whereas neonatal sepsis, neonatal septic work-up, NICU admission and neonatal pneumonia are shown in Figure 3. There was no evidence of heterogeneity in any of the above-mentioned analyses.

Maternal febrile morbidity was reported by two trials,^27^,^28^ both of which showed a nonsignificant reduction (RR 0.94; 95% CI 0.46–1.95; *I*^2^ 0%; forest plot not shown).

Maternal septic thrombophlebitis and maternal pneumonia or respiratory infections were studied in two trials,^24^,^27^ with no events occurring in either trial. Neonatal meningitis was addressed in two trials,^24^,^25^ with no events occurring in either of them. The Thigpen et al. trial^25^ reported one case of neonatal viral syndrome in the preoperative group, which was not significantly different from the intraoperative group (RR 2.9; 95% CI 0.1–71.2).

The length of stay in the NICU in days was reported by three trials.^26^–^28^ There was a significant heterogeneity among the three trials (*I*^2^ = 97%). A sensitivity analysis was performed by omitting the trial with questionable blinding. The degree of heterogeneity improved to an *I*^2^ of 43%. Therefore, this outcome was not considered any further.

### Synthesis of results

Data on endometritis, wound infection, pyelonephritis and total maternal infectious morbidity were available from all six included trials (2313 women); on maternal febrile morbidity from two trials (823 women); on neonatal sepsis, neonatal septic work-up and NICU admission from five trials (1911, 1604 and 2255 newborns, respectively), and on neonatal pneumonia from three trials (1133 newborns).

Preoperative administration of prophylactic antibiotics was associated with a significant 41% reduction in the rate of endometritis (RR 0.59; 95% CI 0.37–0.94) and a significant 29% reduction in the rate of total maternal infectious morbidity (RR 0.71; 95% CI 0.52–0.98) compared with intraoperative administration. In the preoperative administration group, there were nonsignificant reductions relative to the intraoperative administration group in the rates of wound infection (RR 0.71; 95% CI 0.44–1.14), maternal febrile morbidity (RR 0.94; 95% CI 0.46–1.95), neonatal sepsis (RR 0.81; 95% CI 0.47–1.41), neonatal septic work-up (RR 0.93; 95% CI 0.71–1.21) and NICU admission (RR 0.92; 95% CI 0.65–1.28). There was a nonsignificant increase in the rate of maternal pyelonephritis (RR 1.09; 95% CI 0.49–2.43) in the preoperative group. There was also a nonsignificant increase in the rate of neonatal pneumonia (RR 3.36; 95% CI 0.55–20.47) in newborns whose mothers received antibiotics preoperatively. Table 3 shows a summary of the findings, including the quality of the body of evidence, using GradePro.^17^ For all the outcomes reported, the quality of evidence was downgraded to moderate because of imprecision. Imprecision is judged on the bases of optimal information size and the width of the CI. Optimal information size is a sample size calculation to determine whether a trial is sufficiently powered to show a 25% reduction or increase in the risk of the outcome from the median of baseline risks for the included trials. None of the reported outcomes met the optimal information size criterion.

**Table 3:** Summary of findings and quality of the body of evidence for each outcome

Quality assessment							No. of patients		Effect		Quality	Importance
				
No. of studies	Design	Risk of bias	Inconsistency	Indirectness	Imprecisiony	Other considerations	Preoperative	After cord clamping	Relative (95% CI)	Absolute		
**Endometritis (follow up 1–6 weeks)**												
6	Randomised trials	No serious risk of bias	No serious inconsistency	No serious indirectness	Serious^1^	None	27/1158 (2.3%)	47/1155 (4.1%)	RR 0.59 (0.37–0.94)	17 fewer per 1000 (from 2 fewer to 26 fewer)	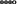 Moderate	Critical^2^
**Wound infection (follow up 1–6 weeks)**												
6	Randomised trials	No serious risk of bias	No serious inconsistency	No serious indirectness	Serious^3^	None	28/1158 (2.4%)	40/1155 (3.5%)	RR 0.71 (0.44–1.14)	10 fewer per 1000 (from 19 fewer to 5 more)	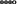 Moderate	Critical^2^
**Pyelonephritis (follow up 1–6 weeks)**												
6	Randomised trials	No serious risk of bias	No serious inconsistency	No serious indirectness	Serious^4^	None	13/1158 (1.1%)	12/1155 (1%)	RR 1.09 (0.49–2.43)	1 more per 1000 (from 5 fewer to 15 more)	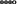 Moderate	Critical^2^
**Neonatal sepsis (follow up 1–6–weeks)**												
5	Randomised trials	No serious risk of bias	No serious inconsistency	No serious indirectness	Serious^5^	None	22/958 (2.3%)	27/953 (2.8%)	RR 0.81 (0.47–1.41)	5 fewer per 1000 (from 15 fewer to 12 more)	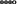 Moderate	Critical^2^
**Neonatal septic work-up (follow up 1–6 weeks)**												
5	Randomised trials	No serious risk of bias	No serious inconsistency	No serious indirectness	Serious^6^	None	94/805 (11.7%)	101/799 (12.6%)	RR 0.93 (0.71–1.21)	9 fewer per 1000 (from 37 fewer to 27 more)	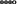 Moderate	Critical^2^
**Neonatal pneumonia (follow up 1–6 weeks)**												
3	Randomised trials	No serious risk of bias	No serious inconsistency	No serious indirectness	Serious^7^	None	4/572 (0.7%)	0/561(0%)	RR 3.36 (0.55–20.47)	–	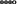 Moderate	Critical^2^
**NICU admission**												
5	Randomised trials	No serious risk of bias	No serious inconsistency	No serious indirectness	Serious^8^	None	60/1126 (5.3%)	66/1129 (5.8%)	RR 0.92 (0.65–1.28)	5 fewer per 1000 (from 20 fewer to 16 more)	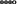 Moderate	Important^2^

1 OIS (Optimal Information Size) of 13256 not met.

2 Patient oriented outcome.

3 OIS of 9444 not met and CI include no effect.

4 OIS of 59370 not met and CI include no effect.

5 OIS of 11796 not met and CI include no effect.

6 OIS of 4292 is not met and CI include no effect.

7 OIS of 67236 is not met and CI include no effect.

8 OIS of 11536 is not met and CI include no effect.

### Risk of bias across studies

As only six trials were included in the meta-analysis, assessment of the possibility of publication bias using a funnel plot and tests of asymmetry are not reported.

### Additional analysis

Because of the possible lack of blinding in the trial carried out by Yildirim et al.,^27^ we conducted a sensitivity analysis of the related outcomes, omitting this trial. The pooled estimate and its 95% CI did not change appreciably in either direction or significance.

## Discussion

### Summary of evidence

While this review was in preparation, another systematic review by Costantine et al.^15^ addressing the same question, which included three randomised trials and two nonrandomised studies, was published in 2008. Three more trials have been published since the publication of that review. Our meta-analysis was discordant in the point estimates but not significantly different from Costantine et al.^15^ for the neonatal septic work-up (their RR 1.0; 95% CI 0.70–1.42, our RR 0.93; 95% CI 0.71–1.21) and NICU admission (their RR 1.07; 95% CI 0.51–2.24, our RR 0.92; 95% CI 0.65–1.28). The numbers of participants in their review for these two outcomes were 771 and 681 newborns, respectively, whereas the corresponding numbers of participants in our review were 1911 and 2255, respectively. Therefore, the discordance is probably attributable to sample size. The effectiveness of prophylactic antibiotics in caesarean section to reduce the risk of maternal infectious morbidity is well established.^1^,^2^ This effectiveness is based on a large number of trials using antibiotic administration after cord clamping. However, the effectiveness of prophylactic antibiotics depends on their presence in effective concentrations throughout the operative period, following administration within 2 hours of surgical incision.^6^ Our review attempted to assess maternal and neonatal infectious outcomes for preoperative administration compared with administration after cord clamping. For maternal outcomes, there was a significant 41% reduction in the rate of endometritis and a significant 29% reduction in the rate of total maternal infectious morbidity when the antibiotic was administered preoperatively. There were nonsignificant reductions in the rates of wound infection and maternal febrile morbidity. For pyelonephritis, there was a nonsignificant increase with preoperative administration. For neonatal outcomes, there were nonsignificant reductions in the rates of neonatal sepsis, neonatal septic work-up and NICU admission, and a nonsignificant increase in the rate of neonatal pneumonia. Although combining similar trials of sufficiently good quality increases the power and precision of the pooled estimate, the number of neonates in the included trials (2345) may not be large enough to allow much confidence to be placed in the apparently reassuring results associated with preoperative administration. The evidence from this review is graded as moderate quality according to the Grade of Evidence Working Group criteria.^30^ A grade of ‘moderate’ means that further research is likely to have an important impact on our confidence in the estimate of effect and may change the estimate. Tita et al.^4^ estimated that as many as 4800 caesarean deliveries would be needed to ascertain a 33% difference in neonatal sepsis with 80% power, assuming a baseline incidence of approximately 5%. However, recent studies on institutional policy changes from administration of antibiotic prophylaxis after cord clamping to preoperative administration confirmed the reduction in maternal infectious morbidity.^31^–^33^ Of these, only the study by Owens et al.^32^ reported on neonatal outcomes. Among 1979 neonates, preincision administration of antibiotics had no adverse effect on neonatal sepsis and neonatal septic work-up.

### Limitations

The number of trials included in our review was small, precluding the construction of meaningful funnel plots and the performance of statistical testing for publication bias, and this may be a limitation of our review.

## Conclusions

Compared with intraoperative administration, preoperative antibiotic administration significantly reduced the rate of endometritis. Other types of infectious maternal morbidity showed lower rates in the preincision administration group, but the differences did not reach statistical significance. Although this review revealed no significant neonatal adverse effects with preincision administration, these results should be interpreted with caution given the power limitation of the included trials.

## Implications for practice

Any consideration of a change in practice based on the evidence presented in this review should take into account the fact that the quality of the evidence for all reported outcomes is moderate.

## Supporting Information

**Appendix S1.** Midline search strategy.
