# Supplementary material for: Timing of administration of prophylactic antibiotics for caesarean section: a systematic review and meta-analysis
Source: BJOG. 2012 Nov 6;120(6):661–9. doi: 10.1111/1471-0528.12036 (PMC3654161; doi:10.1111/1471-0528.12036)
Supplement: Supplementary file 1 [file bjo0120-0661-SD1.pdf]

Appendix S1. Medline search strategy (corresponding MeSH terms were included)

1. Caesarean
2. Caesarean section
3. Caesarean delivery
4. Abdominal delivery
5. 1 or 2 or 3 or 4
6. Antibiotics or antimicrobials
7. Antibiotics or prophylaxis
8. Prophylactic antibiotics
9. 7 and 8
10. 9 and 6
11. 5 and 10

Final results were limited to clinical trials.
